# Development of Leadership Skills in Medical Education: Protocol for a Scoping Review

**DOI:** 10.2196/62810

**Published:** 2024-10-22

**Authors:** Eliana Fazuoli Chubaci, Carlos Dario da Silva Costa, Martins Fideles dos Santos Neto, Emerson Roberto dos Santos, Ana Maria Rita Pedroso Vilela Torres de Carvalho Engel, Ana Caroline dos Santos Costa, Taisa Morete da Silva, Helena Landin Gonçalves Cristóvão, Alex Bertolazzo Quitério, Alba Regina de Abreu Lima, Vânia Maria Sabadoto Brienze, Fernando Nestor Fácio Jr, Júlio César André

**Affiliations:** 1 Center for Studies and Development of Health Education Faculdade de Medicina de São José do Rio Preto São José do Rio Preto, São Paulo Brazil; 2 GEISATEC Research Group Barretos Cancer Hospital Barretos Brazil; 3 Sociedad Latinoamericana de Medicina Sexual SLAMS Rio de Janeiro Brazil

**Keywords:** leadership skills, medical education, undergraduate curriculum, health care management, professional development, scoping review protocol

## Abstract

**Background:**

Leadership is recognized as an essential competency in health care and science, being central for professionals to face health challenges. Few physicians feel prepared to serve as leaders in the health care environment, and few receive training in the leadership skills needed to be successful. Teaching leadership skills together with extensive, longitudinal, clinical education in an authentic and nurturing environment can effectively develop students for leadership in medicine. Studies on the subject still do not show the best way to implement it in medical education, and an updated review is necessary.

**Objective:**

The aim of this study is to identify the types of available evidence on the teaching of leadership skills in undergraduate courses in the health area, analyze them, determine knowledge gaps, and disseminate the research results.

**Methods:**

This is a scoping review that will consider studies on leadership skills in medical and health undergraduate courses. Primary studies published in English, Spanish, and Portuguese since 2019 will be considered. The search will be performed in 8 databases, and reference lists will be searched for additional studies. Duplicates will be removed, and 2 independent reviewers will examine the titles, abstracts, and full texts of the selected studies. Data extraction will be performed using a tool developed by the researchers.

**Results:**

The scoping review is currently in progress. The preliminary database search has been completed, yielding a total of 1213 articles across multiple databases. The next stages, including deduplication, title and abstract screening, and full-text review, are scheduled to be completed by December 2024. Data extraction and analysis are expected to be finalized by March 2025, with the final report anticipated to be ready for submission by June 2025.

**Conclusions:**

This scoping review on leadership in the medical curriculum can significantly contribute to the literature by organizing and synthesizing the available evidence on teaching leadership skills in undergraduate courses in the health area. Furthermore, by analyzing evidence and identifying knowledge gaps, the study can provide valuable insights to develop more efficient and comprehensive medical education programs, thus preparing students to take on leadership roles in the complex environment of health care.

**Trial Registration:**

Open Science Framework YEXKB; https://osf.io/yexkb

**International Registered Report Identifier (IRRID):**

PRR1-10.2196/62810

## Introduction

Leadership is recognized as an essential competency in health care and science [1], being fundamental for professionals to face important health challenges. For this reason, the preparation of health professionals for leadership roles and responsibilities has become increasingly important [2]. Effective leadership through ethical decision-making, communication, teamwork, and flexibility are necessary skills during health crises, having been given greater prominence after the stress inflicted on our health system by the COVID-19 pandemic [3].

There are now more discussions about leadership in medical education. Thus, “medical leadership and management” describe the involvement of physicians in the leadership and management of individual patient care and the departments, organizations, and systems where they work [4]. Therefore, the future of medical leadership and management development has great potential to improve patient care. However, it must be longitudinally integrated throughout physicians’ careers. Furthermore, the development of undergraduate courses is a critical step in increasing awareness, creating professional identity, and thus helping physicians in training to recognize and understand their broader responsibility to the system and patients [4].

The existing gap due to the absence of leadership education leads to the question of whether students develop skills to handle and resolve conflicts, learn to share leadership, prepare others to help and replace them, assume mutual responsibility, and discuss their actions [5]. Informal leadership preparation coupled with extensive, longitudinal, clinical education in an authentic and nurturing environment can effectively develop students for leadership in medicine [6]. Medical knowledge alone is insufficient to provide excellent medical care [7].

In the health care system, leadership has never been more crucial. In low- and middle-income countries, the lack of management capacity leads to frequent failures in initiatives to improve health care, even with clinical knowledge and public health. Some barriers to implementing full leadership development are present at all career levels [8]. Thus, this scoping review aims to fill the following gaps: difficulty in introducing specific programs of leadership training into the medical school curriculum; the existence of curricula filled with other priority topics; and how leaders’ performance should be evaluated during the medical course [9].

Leadership development in medical education is crucial for several reasons:

1. Holistic preparation: The inclusion of leadership in the curriculum helps provide a more holistic and realistic preparation for professional practice and development [3,10,11].
2. Quality of care: Effective leadership is fundamental to generating high-quality care for patients and improving their safety through a collective leadership style [12].
3. Evolving needs: There is an urgent need to adapt current medical curricula, implementing leadership teaching to meet evolving health care needs [1,10,12].
4. Global perspective: It is not known whether medical schools globally promote leadership education and what is the best way to implement it. Therefore, this project is justified to examine leadership education in medical curricula worldwide [4,7,8,12].

Despite the growing effort for physician leadership education to begin earlier and continue throughout their careers [5] and evidence that leadership training must be longitudinally integrated throughout the clinician’s career [12], it is not known whether medical schools globally promote leadership education and what is the best way to implement it. A scoping review can provide a comprehensive, detailed, and unbiased view of this subject. Therefore, a scoping review will be used to address the different perspectives and evidence [4,7,8,12]. The results of this study should allow for a more deep and informed understanding of leadership teaching in medical courses.

Although the benefits of physician leadership are well established according to the *Harvard Business Review* [10], few physicians feel prepared to act as leaders in the complex environment of health care and/or receive training for the leadership skills needed to be successful. Therefore, acquiring leadership should not be different from acquiring the necessary knowledge to become a competent physician [3].

However, studies on the subject greatly differ in methodology, population, context, and objective, making their clear understanding even more difficult. Among all methodologies of knowledge synthesis, scoping reviews are the best way to present a broad view of evidence in heterogeneous scenarios, summarizing and promoting a better understanding [11]. Furthermore, a preliminary search showed that few scoping reviews were found despite the relevance of the topic. How to evaluate leaders’ performance during medical school is also a major gap. In medical school, leaders’ performance appears to be a set of skills different from academic performance, although both types of knowledge can be taught and developed [9]. Therefore, a scoping review on this topic will allow for a more deep and informed understanding of teaching leadership in medical education.

The objective of this scoping review protocol is to identify the types of evidence available on the teaching of leadership skills in undergraduate courses in the health area globally, analyze them, and determine gaps of knowledge.

## Methods

### Overview

The literature search process was conducted using various search databases and strategies in English, Portuguese, and Spanish. Databases included ROAD, MEDLINE, DOAJ, PubMed, Science Direct, Scopus, and Web of Science for the searches in English. For searches in Portuguese, SCIELO was used, whereas for searches in Spanish, SCIELO was also used along with Latindex.

Table 1 summarizes the key components of the search strategy, including the search terms, filters, use of quotations, combination of terms, and an example of an exact search string used in PubMed.

Similar search strings were used for other databases, adapted to their specific syntax and controlled vocabulary.

**Figure 1:**
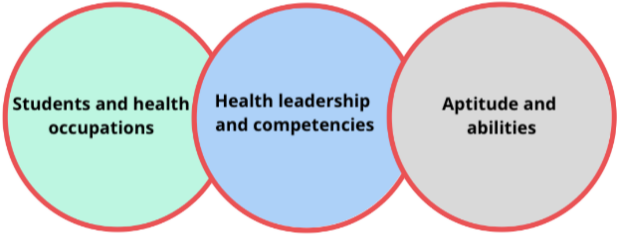
Concepts explained in this review.

The search strategy consisted of combining key terms related to students in the health area, health care leadership competencies, and skills. For searches in English, the terms “Students, Health Occupations” and “Health Leadership, Competencies,” along with the synonyms “Aptitude,” “Abilities,” and their variations, were used. Figure 1 shows the concepts explained in this review.

The research question was formulated in line with the review objective using the mnemonic conceptual model Population, Concept, and Context (PCC): “What is the literature overview regarding leadership skills that can be cultivated during the training of students in the health area?”

**Table 1:** Key components of the search strategy.

Search strategy component	Details
Search terms and keywords	English: “Students, Health Occupations” “Health Leadership, Competencies” “Aptitude” “Abilities” “Aptitudes” “Ability” Portuguese: “Estudantes de Ciências da Saúde” “Competência de Liderança em Saúde” “Aptidão” “Habilidade” “Habilidade Pessoal” Spanish: “Estudiantes del Área de la Salud” “Competencia de Liderazgo en Salud” “Aptitud”
Filters and limits	Date range: Studies published since 2019 Languages: English, Spanish, and Portuguese Publication types: Primary studies, systematic reviews, meta-analyses, meta-syntheses, books, and guidelines published in indexed sources
Use of quotations	Quotation marks were used around specific phrases to ensure exact phrase matching, as shown in the search strategies
Combination of search terms	English: (“Students, Health Occupations”) AND (“Health Leadership, Competencies”) AND (“Aptitude” OR “abilities” OR “Aptitudes” OR “Ability”) Portuguese: (“Estudantes de Ciências da Saúde”) AND (“Competência de Liderança em Saúde”) AND (“Aptidão” OR “Habilidade” OR “Habilidade Pessoal”) Spanish: (“Estudiantes del Área de la Salud”) AND (“Competencia de Liderazgo en Salud”) AND (“Aptitud”)
Exact search string	PubMed: (“Students, Health Occupations”[MeSH^a^ Terms] OR “Students, Health Occupations”[Title/Abstract]) AND (“Health Leadership”[MeSH Terms] OR “Health Leadership”[Title/Abstract] OR “Competencies”[Title/Abstract]) AND (“Aptitude”[MeSH Terms] OR “Aptitude”[Title/Abstract] OR “abilities”[Title/Abstract] OR “Aptitudes”[Title/Abstract] OR “Ability”[Title/Abstract]) AND (“2019/01/01”[Date - Publication] : “3000”[Date - Publication])

^a^MeSH: Medical Subject Headings.

### Inclusion Criteria

For inclusion in this review, studies whose target population was students in the health area will be considered, specifically those addressing leadership skills, regardless of the types of teaching and methodology used to train students.

- Population: Studies whose population consists of medical students and any age groups attending any year of health education will be included.
- Concept: Studies that mentioned “leadership development in medical curricula” will be included, regardless of the type of leadership behaviors and the way the perceptions were measured.
- Context: Eligible studies will be those related to teaching leadership in the health area in any educational context worldwide, regardless of the country of origin of the study.

### Types of Sources

Studies with qualitative and quantitative approaches will be included in the review, including primary studies, systematic reviews, meta-analyses, and/or meta-syntheses, as well as books and guidelines published in indexed sources. For inclusion in this review, opinion, consensus, retraction, editorials, websites, and media advertisement publications will not be considered.

### Exclusion Criteria

The following exclusion criteria will be considered: not meeting the eligibility criteria; not being published in indexed sources; and being publications of opinions, consensuses, retractions, editorials, websites, and advertisements published in the media.

### Search Strategy

In searches in Portuguese, the corresponding terms were the used: “Health Sciences Students”; “Health Leadership Competence”; and “Aptitude,” “Ability,” and their variations. For searches in Spanish, the terms “Estudiantes del Area de la Salud,” “Competencia de Liderazgo en Salud,” and “Apitud” were used (Table 2).

**Table 2:** Search strategy structure.

Category	Details
Subject 1 in Portuguese (DeCS^a^)	“Estudantes de Ciências da Saúde”
Subject 1 in English (MeSH^b^)	“Students, Health Occupations”
Subject 1 in Spanish (DeCS)	“Estudiantes del Área de la Salud”
Subject 2 in Portuguese (DeCS)	“Competência de Liderança em Saúde”
Subject 2 in English (MeSH)	“Health Leadership, Competencies”
Subject 2 in Spanish (DeCS)	“Competencia de Liderazgo en Salud”
Subject 3 in Portuguese (DeCS)	“Aptidão” OR “Habilidade” OR “Habilidade Pessoal”
Subject 3 in English (MeSH)	“Aptitude” OR “abilities” OR “Aptitudes” OR “Ability”
Subject 3 in Spanish (DeCS)	“Aptitud”

^a^DeCS: *Descritores em Ciências da Saúde* (Descriptors in Health Sciences).

^b^MeSH: Medical Subject Headings.

After the question was prepared, the keywords that managed to capture articles referring to the theme of this research were identified: “Knowledge Translation [Title] OR Translational Medical Research [Title/Abstract].” The search was performed using descriptors and/or their synonyms, according to the *Descritores em Ciências da Saúde* (DeCS; Descriptors in Health Sciences) and Medical Subject Headings (MeSH) for each strategy item. To combine descriptors, the AND, OR, and NOT Boolean terms were used.

This diversity of databases and search strategies provided a broad coverage of the available literature, contributing to the comprehensiveness and relevance of the review.

### Study or Source of Evidence Selection

To select studies that meet the inclusion criteria, duplicate articles will initially be removed from the analysis process. Then, 2 researchers will independently analyze the titles and abstracts of the remaining articles using the previously established eligibility criteria. If a disagreement between them occurs regarding the inclusion of a study at this stage, the final decision will be made by a third researcher, who will be consulted to resolve the divergence and determine the relevance of the article to the research question. Additionally, a manual search for relevant sources not captured in the initial search strategy may complement the review, as long as they meet the eligibility criteria and significantly contribute to the conclusion of the study.

Next, the main researcher will evaluate the full texts of the previously selected articles, ensuring that they are aligned with the inclusion criteria. The reasons for excluding any studies after full-text reading will be appropriately documented and presented in the context of the scoping review. At any stage of the selection process, any disagreements that may arise between members of the research team will be resolved either through a consensual discussion or with the intervention of additional researchers to reach a consensus if necessary.

The study selection process will be reported using a PRISMA (Preferred Reporting Items for Systematic Reviews and Meta-Analyses) flow diagram to ensure transparency and reproducibility.

### Data Extraction

After the article selection phase, the main researcher will prepare a form for data extraction, which will be completed after a thorough analysis of each selected article. The data to be extracted will cover specific information related to the PCC and methodology of the study, in addition to relevant data for the research question such as method and model of leadership teaching adopted, strategies used, perceptions of the studied population, and identified impact. The initial draft of the form is presented in Multimedia Appendix 1 and can be adapted and improved as necessary during the extraction of data from each bibliographic source included in the scoping review. Any adjustments made will be duly documented in the revision scope. Furthermore, the article authors will be contacted to clarify missing information when applicable or obtain additional data to ensure the integrity and accuracy of data analysis. The data extraction indicators are presented in Table 3.

**Table 3:** Data extraction indicators developed by the researchers.

Category	Indicator
Publication details	Journal Year Title Authors Country Type of study
Inclusion or exclusion criteria	Population Concept Context Reason for exclusion
Findings	Leadership teaching method Leadership teaching model Student perception Impact

### Analysis of the Evidence

The analysis of the results will be conducted following the objectives established in this scoping review. As is typical for scoping reviews, we will provide a narrative synthesis of the findings, mapping the available evidence on leadership development in medical education. Descriptive statistics such as absolute frequencies and percentages will be used to summarize characteristics of publications (journal, year, title, authors, country, and type of study). However, unlike a systematic review, we will not conduct in-depth quantitative analyses or assess the quality of individual studies. We will use a qualitative content analysis approach to explore and categorize findings related to the method and model of teaching leadership, student perception, and identified impact. This analysis will focus on identifying patterns and themes across the included studies, rather than synthesizing effectiveness data as would be done in a systematic review. Data will be presented in figures and tables, complemented by a narrative synthesis to describe the relationship of the results to the research question. This approach aligns with the purpose of a scoping review, which is to map the available evidence and identify knowledge gaps, rather than to answer a specific clinical question or assess the quality of evidence as in a systematic review. The results will be reported following the PRISMA-ScR (PRISMA Extension for Scoping Reviews) guidelines to ensure transparency and reproducibility of our scoping review process.

The complete search strategies used for each database are provided in Multimedia Appendix 2.

## Results

The scoping review is currently in progress. The preliminary database search has been completed, yielding a total of 1213 articles across multiple databases. The next stages, including deduplication, title and abstract screening, and full-text review, are scheduled to be completed by December 2024. Data extraction and analysis are expected to be finalized by March 2025, with the final report anticipated to be ready for submission by June 2025.

## Discussion

### Expected Findings

The recognized needs for effective succession planning and leadership training are well established, with a current shortage of emerging leaders assuming leadership roles [5,13,14]. Effective leaders require support and guidance from the organizations where they were educated and trained, and where they work [15]. Leadership as a learned skill is gaining momentum as a fundamental curriculum item in medical education. Leadership development, assessment, and feedback are essential throughout the education and training of health professionals. Aspiring and current leaders can be identified, trained, and assessed through formal leadership development programs and organizational supportive cultures. This requires the incorporation of programs for leadership training, opportunities for leadership practice, and promotion of professional networks within and outside the organization. Mentoring in health education is recognized and important, and it offers a means to further enhance leadership and engagement in the workforce [16,17].

Leadership is composed of a set of practices and skills that can be learned and can be developed by reading literature articles and participating in leadership courses [17]. Moreover, investment in the social capital of organizations, promoting interprofessional learning and communication in the workplace, and collaboration between organizations help develop leadership. The development of leadership skills is a lifelong process [18,19]. Resources and opportunities should be considered to help develop leadership skills through activities such as reading about leadership theories, attending leadership training workshops, engaging in mentoring programs, attending small-group seminars on leadership development, and accepting more responsibilities when necessary or when opportunities arise [20].

Effective leadership does not necessarily require an academic title. It can manifest itself in daily work and collaboration with other professionals in education and health systems, such as teaching, administration, research, and clinical practice [3]. Leadership functions include the important concepts of managing personal and professional practices, prioritizing tasks, time management, and effective delegation [21].

Research indicates that while students consider leadership an essential competency due to the responsibilities inherent to the medical profession, professors may not see the need to separately consider leadership as an essential competency due to the implicit training provided by the curriculum. However, both students and professors identify rigorous curricula as barriers to implementing leadership training. Students also highlight the feasibility of leadership training as a challenge, especially in clinical settings. However, the lack of recognition seems to be the most influential obstacle [22-24].

Qualities such as emotional intelligence, confidence, and humility can be taught during medical courses along with skills in teamwork, communication, and management. As a result, medical institutions have introduced explicit leadership curricula and integrated leadership training into their programs to meet these needs [25].

The consistent themes behind the implementation of curricula and explicit leadership structures in medical schools emphasize the intention to actively embody and pursue leadership skills and competencies that can be naturally observed and taught. Integrating training with these principles in mind would be much more effective against the obstacles posed by overloaded curricula and would increase recognition or at least reduce disinterest in leadership training [26].

Implementing changes based on a curriculum model would be beneficial, as it is described as one of the most comprehensive models for leadership education. However, incorporating leadership as a core objective and constructing or adapting a conceptual framework for medical curricula similar to the examples mentioned above, while considering student perceptions, would be prudent. Incorporating such training would be more effective if done through experiential learning, although additional small-group sessions and classroom-based interactive seminars could also be used [27].

Finally, the methods used in residency training to develop leadership can be extended to medical schools. Providing formative feedback on leadership skills can be effective in improving leadership skills. Ultimately, training must be integrated and aligned with education rather than separated and isolated from clinical instruction. Curriculum planners must be flexible and engage with medical professionals to emphasize the importance of such a competency [28].

### Limitations

The limitation of this research is the possible incompleteness or bias of the available data sources, although an extensive search was conducted in several databases. Although the inclusion and exclusion criteria have been carefully defined, omission or nonidentification of some relevant studies during the search is possible. Due to the comprehensive nature of the scoping review, it may also not allow for a detailed analysis of each study, thus limiting the depth of assessment of the available evidence.

To mitigate these limitations, we will implement the following strategies:

1. Collaborate with an experienced librarian to refine our search strategy, ensuring comprehensive coverage across databases
2. Include gray literature sources such as conference proceedings and organizational reports to capture non–peer-reviewed but relevant information
3. Conduct hand-searching of reference lists from the included studies and relevant systematic reviews
4. Consult with experts in medical education and leadership to identify key studies that may have been overlooked
5. Maintain transparent reporting of all methodological decisions and processes

Another significant limitation is the lack of clarity on how leaders’ performance should be evaluated during medical school. To address this gap, our review will specifically focus on identifying and synthesizing information about evaluation methods, including but not limited to the following:

- Multisource feedback mechanisms
- Objective Structured Leadership Examinations
- Leadership portfolios
- Longitudinal assessment approaches
- Self-assessment tools
- Project-based assessments

For a more comprehensive and precise understanding, it is important to recognize that the interpretation of the results of this review must consider these possible limitations and be complemented by other sources of evidence when necessary. We will explicitly discuss these limitations in our final report and suggest areas for future research to address remaining gaps in knowledge.

These revisions address the potential biases and limitations of the study, as well as the gap in understanding how to evaluate leadership performance in medical education.

### Conclusions

In this research protocol, a scoping review on the development of leadership skills in medical education was outlined, recognizing the fundamental importance of leadership in clinical practice and health sciences. The proposed review aims to address the existing gap in the literature on the implementation of leadership education in health care colleges by highlighting the need to prepare students to assume leadership roles in the complex environment of health care. The preliminary results show a wide range of articles, pointing out the relevance and urgency of this topic. This review offers valuable insights to develop more comprehensive and effective educational programs, empowering students to lead interdisciplinary teams and promote positive change in the health system.

Furthermore, the inclusion of leadership in the medical courses curriculum can provide more holistic and realistic preparation for future health care professionals, equipping them with the necessary skills to provide high-quality care to patients and face emerging challenges in the health area. This scoping review will not only contribute to the literature but will also have a practical and tangible impact on students professional training and development, thus promoting significant advances in medical practice and education.

## Appendix

Multimedia Appendix 1 Structuring search performance.

Multimedia Appendix 2 Detailed search strategies for each database used in the scoping review.

Multimedia Appendix 3 PRISMA-P (Preferred Reporting Items for Systematic Review and Meta-Analysis Protocols) checklist.

## Abbreviations

DeCS: Descritores em Ciências da Saúde (Descriptors in Health Sciences)
MeSH: Medical Subject Headings
PCC: Population, Concept, and Context
PRISMA: Preferred Reporting Items for Systematic Reviews and Meta-Analyses
PRISMA-ScR: Preferred Reporting Items for Systematic Reviews and Meta-Analyses Extension for Scoping Reviews

## Data Availability

All data generated or analyzed during this scoping review will be included in the published article and its supplementary files. The complete list of included studies, along with their extracted data, will be made available as a supplementary file upon publication. The search strategies for each database are provided in Multimedia Appendix 2. Any additional data related to this study are available from the corresponding author upon reasonable request.
