# Supplementary material for: Development of Leadership Skills in Medical Education: Protocol for a Scoping Review
Source: JMIR Res Protoc. 2024 Oct 22;13:e62810. doi: 10.2196/62810 (PMC11538880; doi:10.2196/62810)
Supplement: Multimedia Appendix 1 [file resprot_v13i1e62810_app1.docx]

Table 1. Key components of the search strategy.

| **Search Strategy Component** | **Details** |
| --- | --- |
| Search Terms and Keywords | **English:**  - "Students, Health Occupations"  - "Health Leadership, Competencies"  - "Aptitude"  - "Abilities"  - "Aptitudes"  - "Ability"   **Portuguese:**  - "Estudantes de Ciências da Saúde"  - "Competência de Liderança em Saúde"  - "Aptidão"  - "Habilidade"  - "Habilidade Pessoal"   **Spanish:**  - "Estudiantes del Área de la Salud"  - "Competencia de Liderazgo en Salud"  - "Aptitud" |
| Filters and Limits | - Date range: Studies published since 2019  - Languages: English, Spanish, and Portuguese  - Publication types: Primary studies, systematic reviews, meta-analyses, meta-syntheses, books, and guidelines published in indexed sources |
| Use of Quotations | Quotation marks were used around specific phrases to ensure exact phrase matching, as shown in the search strategies |
| Combination of Search Terms | **English:**  ("Students, Health Occupations") AND ("Health Leadership, Competencies") AND ("Aptitude" OR "abilities" OR "Aptitudes" OR "Ability")   **Portuguese:**  ("Estudantes de Ciências da Saúde") AND ("Competência de Liderança em Saúde") AND ("Aptidão" OR "Habilidade" OR "Habilidade Pessoal")   **Spanish:**  ("Estudiantes del Área de la Salud") AND ("Competencia de Liderazgo en Salud") AND ("Aptitud") |
| Exact Search Strings | **PubMed:**  ("Students, Health Occupations"[MeSH Terms] OR "Students, Health Occupations"[Title/Abstract]) AND ("Health Leadership"[MeSH Terms] OR "Health Leadership"[Title/Abstract] OR "Competencies"[Title/Abstract]) AND ("Aptitude"[MeSH Terms] OR "Aptitude"[Title/Abstract] OR "abilities"[Title/Abstract] OR "Aptitudes"[Title/Abstract] OR "Ability"[Title/Abstract]) AND ("2019/01/01"[Date - Publication] : "3000"[Date - Publication]) |

Source: Authors, 2024.
