# Supplementary material for: Development of Leadership Skills in Medical Education: Protocol for a Scoping Review
Source: JMIR Res Protoc. 2024 Oct 22;13:e62810. doi: 10.2196/62810 (PMC11538880; doi:10.2196/62810)
Supplement: Multimedia Appendix 2 [file resprot_v13i1e62810_app2.docx]

Multimedia Appendix 2: Detailed search strategies for each database used in the scoping review.

| DATABASE | STRATEGY |
| --- | --- |
| Search Strategies IN ENGLISH:  **- Directory of Opens Access Scholary Resources (ROAD); Medline; Directory of Open Access Journal (DOAJ; Pubmed; Science Direct; SCOPUS and Web of Science.** | (“Students, Health Occupations”) and (“Health Leadership, Competencies” ) AND (“Aptitude” OR “abilities” OR “Aptitudes” OR “Ability”) |
| Search Strategies IN PORTUGUESE:  **Scientific Electronic Library Online** **(SCIELO)** | (“Estudantes de Ciências da Saúde”) AND  (“Competência de Liderança em Saúde”) AND  (“Aptidão” OR “Habilidade” OR “Habilidade Pessoal”) |
| Search Strategies IN PORTUGUESE:  **Scientific Electronic Library Online** **(SCIELO)**; **Latindex** | (“**Estudiantes del Área de la Salud) AND**  (“Competencia de Liderazgo en Salud”) AND (“Apitud”) |
